# Turning behaviors of T cells climbing up ramp-like structures are regulated by myosin light chain kinase activity and lamellipodia formation

**DOI:** 10.1038/s41598-017-11938-y

**Published:** 2017-09-14

**Authors:** Kwang Hoon Song, Jaehyun Lee, Hong-Ryul Jung, HyoungJun Park, Junsang Doh

**Affiliations:** 1 Department of Mechanical Engineering, Pohang University of Science and Technology (POSTECH) San 31, Hyoja-dong, Nam-Gu, Pohang, Gyeongbuk 790-784 Korea; 2 School of Interdisciplinary Bioscience and Bioengineering (I-Bio), Pohang University of Science and Technology (POSTECH) San 31, Hyoja-dong, Nam-Gu, Pohang, Gyeongbuk 790-784 Korea; 3 Present Address: Department of Bioengineering, University of Pennsylvania, 210 South 33rd Street, Philadelphia, PA 19104 USA

## Abstract

T cells navigate diverse microenvironments to perform immune responses. Micro-scale topographical structures within the tissues, which may inherently exist in normal tissues or may be formed by inflammation or injury, can influence T cell migration, but how T cell migration is affected by such topographical structures have not been investigated. In this study, we fabricated ramp-like structures with a 5 μm height and various slopes, and observed T cells climbing up the ramp-like structures. T cells encountering the ramp-like structures exhibited MLC accumulation near head-tail junctions contacting the ramp-like structures, and made turns to the direction perpendicular to the ramp-like structures. Pharmacological study revealed that lamellipodia formation mediated by arp2/3 and contractility regulated by myosin light chain kinase (MLCK) were responsible for the intriguing turning behavior of T cells climbing the ramp-like structures. Arp2/3 or MLCK inhibition substantially reduced probability of T cells climbing sharp-edged ramp-like structures, indicating intriguing turning behavior of T cells mediated by lamellipodia formation and MLCK activity may be important for T cells to access inflamed or injured tissues with abrupt topographical changes.

## Introduction

T cells are immune cells in adaptive immunity responsible for the initiation and orchestration of antigen-specific immune responses. T cells migrate throughout the body to perform immune surveillance and to mount immune responses against pathogens and tumors^1, 2^. To efficiently survey large areas of tissues and organs, T cells utilize a number of strategies^3, 4^: they exert fast motility, about 100-fold faster than that of typical mesenchymal cells^5^, with seemingly random motility^6, 7^ described by modified forms of random walks such as persistent random walk or Levy walk^8^. At the same time, their migration is frequently guided by not only chemokines, but also various tissue structures including fibrillary structures^9, 10^, vasculatures^11, 12^, and stromal cell networks^13^, which is likely to bring T cells to anatomically or topologically distinct locations of tissues with an enhanced probability of finding targets^14–16^.

Biochemical signals presenting on tissue structures, such as adhesion molecules and surface-bound chemokines, can direct the adhesion and migration of T cells. Alternatively, the distinct micro/nanoscale topographical structure of the tissue itself can serve as a biophysical cue guiding motility^17–19^. Microfabricated surfaces presenting various topographical structures can be a powerful tool to investigate how surface topography regulates cell migration by allowing the independent control of surface topography and chemistry^20, 21^. Using this strategy, we fabricated periodic structures of nanoscale groove/ridge patterns^22, 23^, which mimic the topography of extracellular matrixes (ECMs), or sinusoidal wavy structures with wavelengths of tens of micrometers^24, 25^, which mimic the topography of cell monolayers or curvatures of vasculatures, and systematically investigated how T cells sense and respond to various topographical structures.

In this study, ramp-like structures of ~5 μm in height were fabricated and the behaviors of T cells encountering and climbing up the ramp-like structures were studied by video microscopy. The ramp-like structure used in the study is rather artificial, but such gradual changes in topography may occur *in vivo* near the interfaces between tissues or tissue compartments. Interestingly, T cells climbing up the ramp-like structures frequently turned to the perpendicular direction of the ramp-like structures. The molecules responsible for this intriguing turning behavior were further identified and characterized by pharmacological inhibitors and fluorescence live-cell imaging.

## Results

### Fabrication of various ramp-like structures for T cell migration study

**Figure 1:**
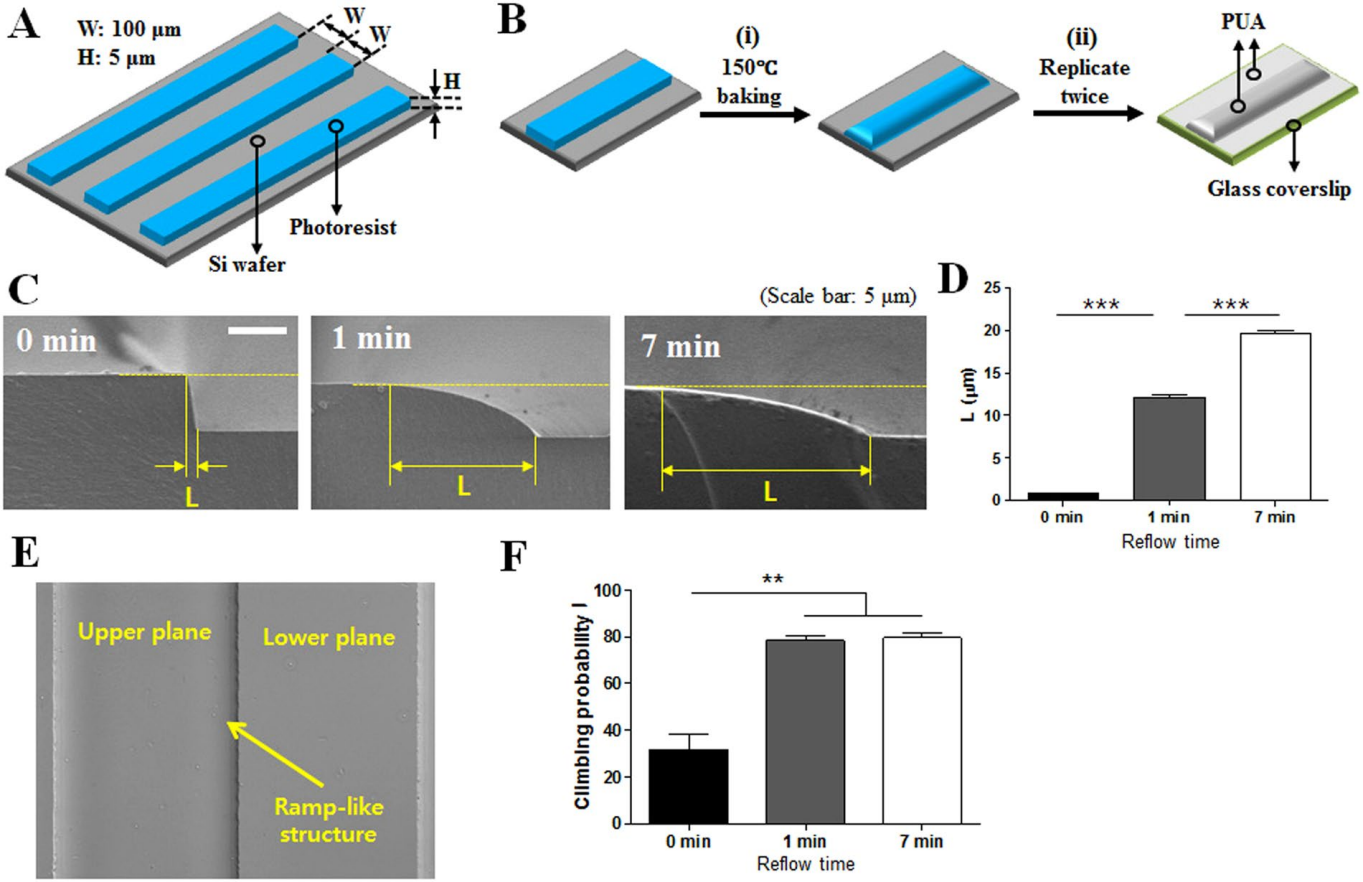
Fabrication schemes and experimental settings of ramp-like structures for T cell migration study. (**A**) A schematic illustration of periodic photoresist stripe patterns on silicon wafers fabricated by a standard photolithography technique. (**B**) Fabrication scheme of the ramp-like structures. (i) Smoothening photoresist stripe edges by heat, and (ii) replicating the ramp-like structures onto the glass coverslips using UV-curable resin poly(urethane acrylate) (PUA). (**C**) Cross-sectional scanning electron microscope (SEM) images of the ramp-like structures fabricated with various baking times. Scale bar: 5 μm. (**D**) Average length (L) of the ramp-like structures fabricated with various baking times. (Unpaired T test, two-tailed, ***p < 0.001). (**E**) Differential interference contrast (DIC) images of the ramp-like structures at the interface of upper and lower planes. Baking time: 1 min. (**F**) Probability of T cells climbing the ramp-like structures fabricated with various baking times. 0 min: *n* = 110; 1 min: *n* = 144; 7 min: *n* = 141. (Mann-Whitney U-test, two-tailed, **p < 0.01).

To fabricate smooth ramp-like structures, first, periodic stripe patterns of a photoresist polymer of 100 μm in width, 5 μm in height with a 100 μm interval were fabricated onto flat silicon wafers by a standard photolithography technique (Fig. 1A). By baking the patterned wafers at 150 °C, reflow of photoresist patterns near the sharp edges of the stripes occurred to form smooth ramp-like structures (Fig. 1B-(i)). The ramp-like structures on glass coverslips were obtained by replicating the ramp-like structures fabricated on the silicon wafer twice by capillary force lithography (CFL)^26^ using UV-curable resin polyurethane acrylate (PUA) (Fig. 1B-(ii)). Cross-sectional scanning electron microscopy (SEM) images of the successfully fabricated ramp-like structures with various baking times are shown in Fig. 1C. Increasing the baking time significantly increased the length of the ramp-like structure (L) by enhancing reflow of the polymeric photoresist (Fig. 1D), resulting in the smoothening of sharp edges. The interfaces between the ramp-like structures and lower planes fabricated on glass coverslips are clearly visible on differential interference contrast (DIC) images obtained by optical microscopy (Fig. 1E). Similar to previous experiments examining the effects of surface topography on T cell migration^22, 23, 25^, PUA ramp-like structures were coated with Intercellular Adhesion Molecule 1 (ICAM-1) to support the firm adhesion and migration of T cells. T cells were seeded on ICAM-1-coated PUA ramp-like structured surfaces treated with various baking times (0, 1, and 7 min) and observed by video microscopy. While ~70% of T cells encountering sharp-edged ramp-like structures (or surfaces with 0 min of baking) from the lower planes failed to climb ramp-like structures, ~80% of T cells encountering smooth-edged ramp-like structures (or surfaces with 1 or 7 min of baking) from the lower planes successfully climbed to the upper planes (Fig. 1F). Considering that the behaviors of the T cells were almost identical between ramp-like structures with 1 and 7 min of baking and the non-physiological sharp-edges of ramp-like structures with 0 min of baking, the ramp-like structures with 1 min of baking were used for the bulk of the experiments unless otherwise mentioned.

### T cells climbing up ramp-like structures maintain constant turning angles regardless of the approaching angles

**Figure 2:**
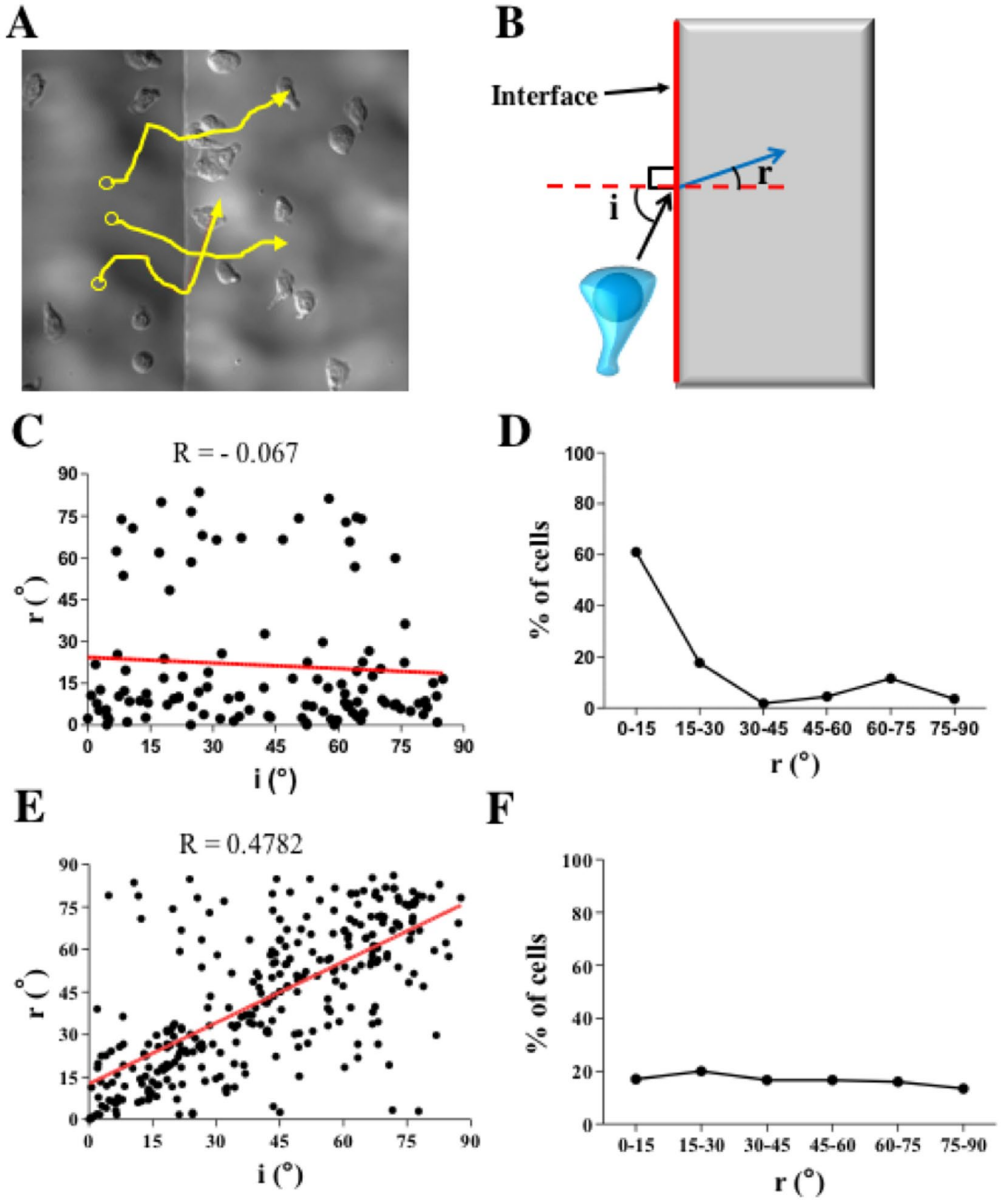
Migration direction analysis of T cells climbing up the ramp-like structures. (**A**) Representative trajectories of T cells climbing up the ramp-like structures, overlaid on a DIC image. (**B**) Definition of the incidence (i) and refraction (r) angles of T cells climbing up the ramp-like structures. (**C**,**D**) Correlation between the incidence and refraction angles (**C**), and distribution of refraction angles (**D**) of T cells climbing the ramp-like structures. *n* = 144. (**E**,**F**) Correlation between the incidence and refraction angles (**E**), and distribution of refraction angles (**F**) of T cells crossing an imaginary line on flat surfaces. *n* = 305.

To analyze the behaviors of T cells climbing up the ramp-like structures, time-lapse DIC images of T cells were acquired over 20 min. The trajectories of T cells were extracted from the time-lapse images and overlaid with the last still image of the time-lapse images (Fig. 2A). Frequently, T cells crawling up the ramp-like structures changed their direction when they crossed interfaces between lower planes and ramp-like structures. The changes in the directions of T cells crossing the interfaces of lower planes and ramp-like structures were quantitatively analyzed by the method used to characterize the refraction of light, as schematically shown in Fig. 2B, by defining the incidence angle ‘i’ and refraction angle ‘r’. To find a correlation between i and r, we plotted the i *vs*. r values of individual T cells (144 cells in total) (Fig. 2C). Surprisingly, there was no correlation between the incidence angle and refraction angle (R ~ 0; 0 < R < 1 for positive correlation and −1 < R < 0 for negative correlation), and the majority of T cells exhibited r < 30°, while a small fraction of T cells exhibited r > 60° (and almost no T cells were found in 30° < r < 60°) regardless of i values, meaning most of the T cells crossing ramp-like structures turned perpendicular to the interfaces, while a small fraction of T cells turned parallel to the interfaces. This tendency was confirmed by analyzing the distribution of refraction angles (Fig. 2D): ~60% of T cells were in r < 15°, and ~80% of T cells were in r < 30°, while ~10% of T cells were located in the second peak, where 60° < r < 75°. In sharp contrast, when we drew an imaginary line on the lower plane and performed the same analysis for T cells crossing the imaginary line, incidence and refractory angles exhibited positive correlation (R = 0.4782; Fig. 2E), and refraction angle exhibited uniform distribution (Fig. 2F).

### Lamellipodia formation and myosin light chain kinase (MLCK) activity regulated turning of T cells climbing ramp-like structures

**Figure 3:**
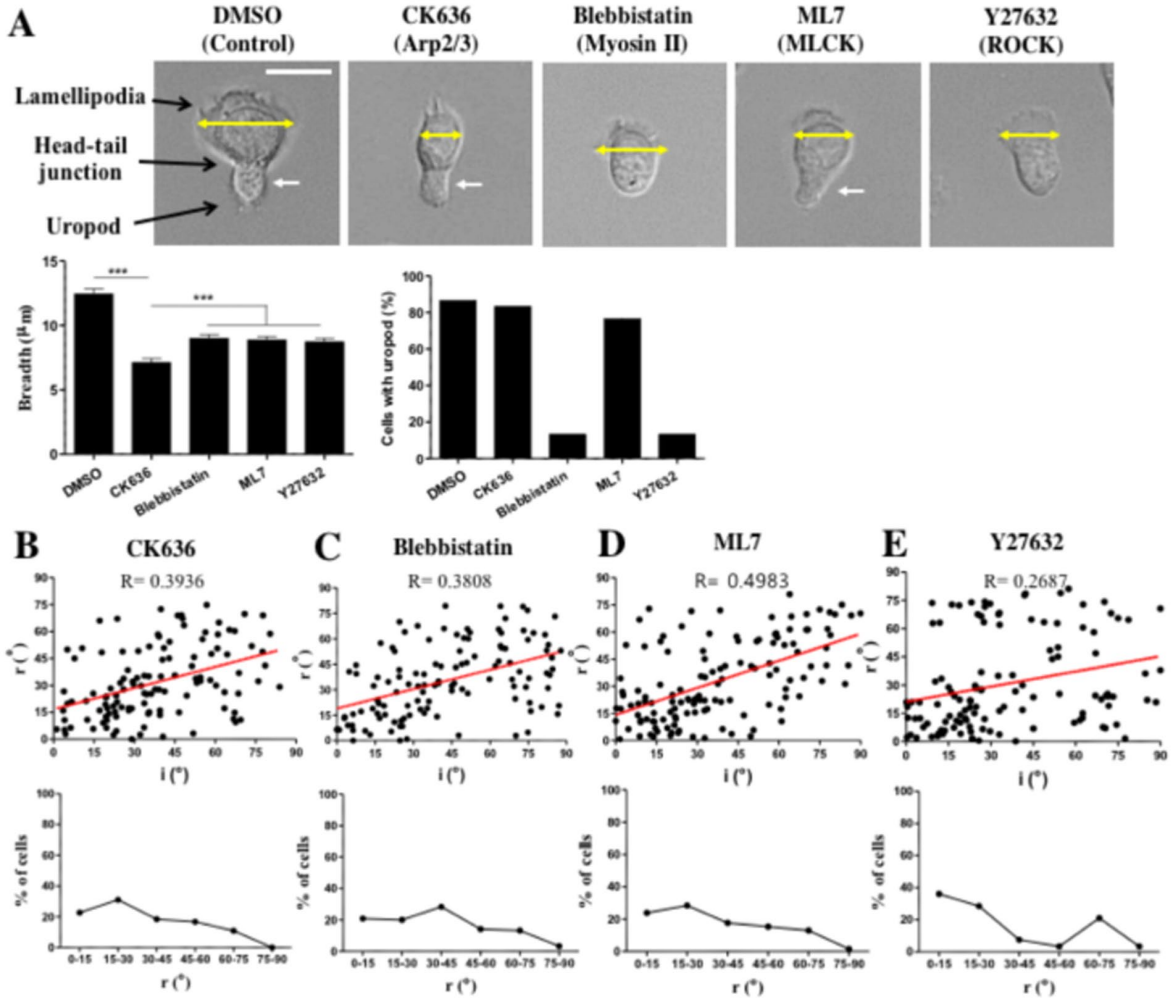
The effects of pharmacological inhibitors on the turning behaviors of T cells encountering the ramp-like structures. (**A**) DIC images (upper panels) and morphology analysis (lower panels) of inhibitor-treated T cells. Yellow arrow: breadth of lamellipodia, white arrow: uropod. *n* = 30 for each condition. Scale bar: 10 μm. (**B**–**E**) Correlations between the incidence and refraction angles (upper panels) and distributions of refraction angles (lower panels) of CK636- (**B**), Blebbistatin- (**C**), ML7- (**D**) and Y27632- (**E**) treated T cells climbing up the ramp-like structures. CK636: *n* = 119; Blebbistatin: *n* = 120; ML7: *n* = 130; Y27632: *n* = 119.

To identify the molecules involved in this intriguing turning behavior of T cells encountering the ramp-like structures, we first inhibited actin-related protein-2/3 (Arp2/3) complex-mediated lamellipodia formation at the leading edges and myosin II-mediated contractility, which have been shown to be important for topography-guided migration of T cells^22, 25^, by treating T cells using pharmacological inhibitors CK636 and blebbistatin, respectively. As previously reported, CK636-treated T cells exhibited sharp leading edges^22, 25^ and blebbistatin-treated T cells lacked uropods^25, 27^ (Fig. 3A). The turning angles of inhibitor-treated T cells climbing up the ramp-like structures were analyzed as described previously (upper panels of Fig. 3B and C). Unlike untreated T cells (Fig. 2C), the incidence angles and refraction angles of T cells treated with CK636 and blebbistatin exhibited positive correlations with R values of 0.3936 and 0.3808, respectively, meaning that the migration directions of CK636 or blebbistatin-treated T cells climbing up the ramp-like structures were relatively well conserved compared with those for untreated T cells. In addition, relatively uniform distributions of refraction angles were obtained for both inhibitor-treated T cells (lower panels of Fig. 3B and C). These results indicate that arp2/3-mediated lamellipodia formation and myosin II-mediated contractility are critical for the turning of T cells climbing up the ramp-like structures.

Myosin II activities in T cells are differentially regulated by two distinct kinases, myosin light chain kinase (MLCK) and Rho-associated protein kinase (ROCK)^28^. To further identify the kinases responsible for the turning behaviors of T cells climbing ramp-like structures, T cells were treated with ML7, which inhibits MLCK, and Y27632, which inhibits ROCK. Similar to blebbistatin-treated T cells, ML7-treated T cells exhibited a positive correlation between incidence and refraction angles and the relatively uniform distribution of refraction angles (Fig. 3D). In contrast, the behaviors of Y27632-treated T cells (Fig. 3E) were closer to those of the control T cells (Fig. 2C and D), considering the relatively weak correlation between incidence angles and refraction angles and two peaks at r < 15° and 60° < r < 75° in refraction angle distribution (Fig. 3F). These results suggest that MLCK-mediated contractility is more responsible for the turning of T cells climbing the ramp-like structures than ROCK-mediated contractility.

### Accumulation of myosin light chain near head-tail junctions of T cells determined turning directions

**Figure 4:**
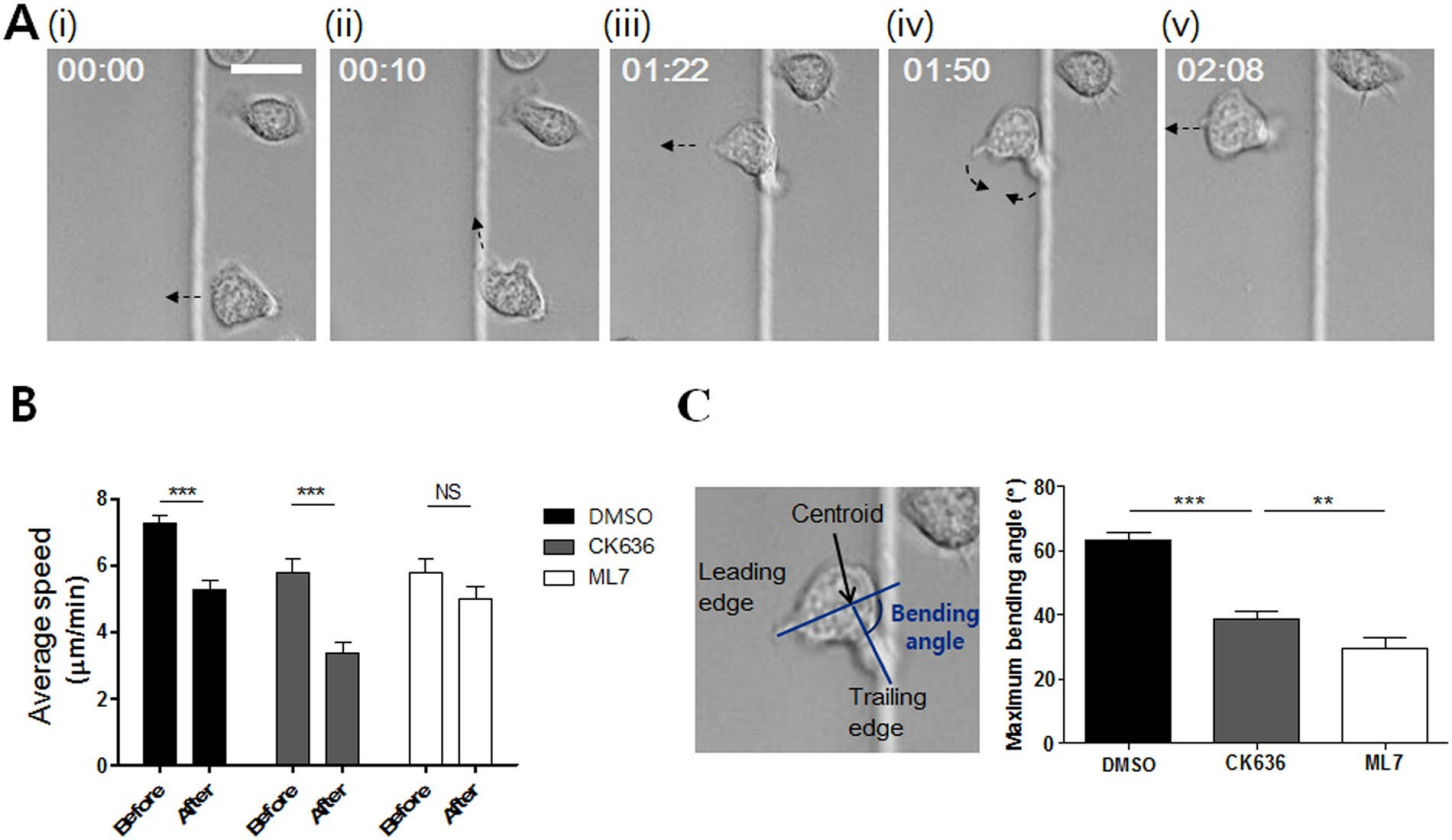
Detailed behavior analysis of T cells encountering the ramp-like structures. (**A**) Representative time-lapse DIC images of a T cell climbing up the ramp-like structure. Elapse time: mm:ss. Scale bar: 10 μm. (**B**) Average crawling speed of T cells for 1 min “before” and “after” encountering the ramp-like structures. *n* = 10 for each condition. (**C**) Definition of bending angles between trailing and leading edges (left panels) and the average of maximum bending angles (right panels) of T cells during climbing up the ramp-like structure. *n* = 30 for each condition. (Mann-Whitney U-test, two-tailed, **p < 0.01, ***p < 0.001).

The pharmacological inhibitor study clearly demonstrated that the Arp2/3 complex-mediated lamellipodia formation and MLCK-mediated myosin II activities play critical roles in the turning behaviors of T cells climbing ramp-like structures. Next, we performed high-temporal-resolution DIC live cell imaging to gain further information about detailed behaviors of T cells climbing ramp-like structures (Fig. 4A and Movie S1 in Supplementary Information (SI)). Frequently, T cells encountering the ramp-like structures first turned to the direction parallel to the interfaces of the ramp-like structures (Fig. 4A(ii)). T cells then stretched out their leading edges perpendicular to the interfaces (Fig. 4A(iii)), bent their mid-bodies for swift turns (Fig. 4A(iv)), and finally crawled perpendicular to the interfaces (Fig. 4A(v)). T cells encountering the ramp-like structures significantly reduced their migration speed as assessed by the average speed for 1 min before and after encountering the ramp-like structures (Fig. 4B). CK636-treated T cells exhibited a similar reduction in velocity even though they did not change direction while climbing up. In contrast, the velocity of ML7-treated T cells was almost constant while climbing up the ramp-like structures. These results suggest that MLCK-mediated contractility is responsible for the reduction in velocity upon encountering the ramp-like structures. The bending angles between trailing edges and the leading edges of T cells climbing the ramp-like structures were measured, and the maximum bending angle of a T cell during climbing the ramp-like structure was selected and plotted (Fig. 4C). While untreated T cells substantially bent their cell bodies for swift turns, CK636-treated or ML7-treated T cells minimally bent their cell bodies during climbing the ramp-like structures. Notably, the average maximum bending angle of CK636-treated T cells was significantly greater than that of ML7-treated T cells, indicating that MLCK-mediated contractility is a major driving force for the swift turning of T cells that encounter ramp-like structures. Taken together, MLCK activity was responsible for the reduction of velocity and turning of T cells encountering the ramp-like structures.

**Figure 5:**
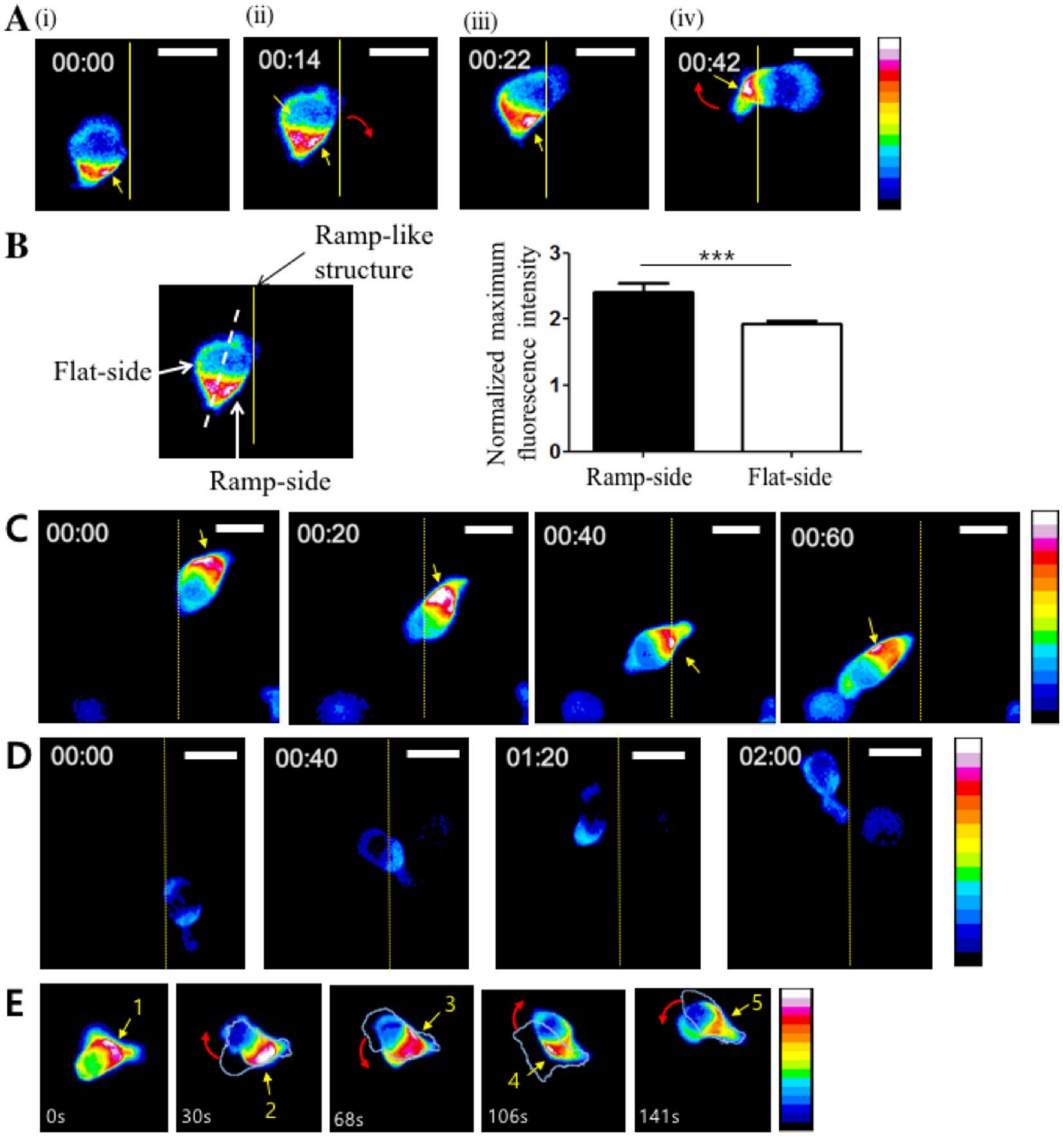
Distribution of myosin light chain (MLC) in T cells climbing up the ramp-like structures visualized by time-lapse images of MLC-GFP. (**A**) Pseudo-color time-lapse images visualizing the relative fluorescence intensities of MLC-GFP in DMSO-treated T cells climbing up the ramp-like structures. Elapse time: mm:ss. Scale bar: 10 μm. (**B**) Comparison of maximum fluorescence intensity between ramp-side and flat-side of T cells climbing up the ramp-like structure. T cells just before swift turns were analyzed, thus most of T cells analyzed exhibited relatively straight morphology without substantial bending. (**C**–**D**) Pseudo-color time-lapse images visualizing the relative fluorescence intensities of MLC-GFP in CK636- (**C**) and ML7- (**D**) treated T cells climbing up the ramp-like structures. Elapse time: mm:ss. Scale bar: 10 μm. (**E**) Pseudo-color time-lapse images visualizing the relative fluorescence intensities of MLC-GFP in a T cell crawling on flat PUA surfaces. Boundaries of T cells in the previous time point are overlaid with white lines. Yellow arrow: the highest intensity of MLC-GFP in each image. Red arrow: Turning direction.

The phosphorylation of MLC by MLCK triggers the bundling of myosin II and actomyosin assembly, resulting in the local accumulation of myosin II and generation of contractile force^29^. To visualize myosin II dynamics in T cells during climbing ramp-like structures, MLC-GFP was expressed in T cells and time-lapse imaging of MLC-GFP was performed every 2 sec. Pseudo-color time-lapse images visualizing the relative fluorescence intensities of MLC-GFP in T cells in each time frame are shown in Fig. 5A and Movie S2 in SI. The interfaces between lower planes and ramp-like structures are drawn as yellow lines. Consistent with the previous observation using MyH9-GFP^27^, dynamic packets of MLC-GFP were frequently observed in head-tail junction regions (depicted in Fig. 3A) for most of the T cells climbing the ramp-like structures (30 out of 36 cells). Notably, MLC-GFP packets often localized to the side of the head-tail junction contacting the ramp-like structures (Fig. 5A(i–iii)) prior to swift turns to the direction perpendicular to the interfaces (Fig. 5A(iii,iv)). To quantitatively analyze this tendency, a T cell contacting the ramp-like structure was divided into half, maximum fluorescence of the T cell contacting the ramp-side was compared with that of the T cell on the flat-side just before turning (Fig. 5B). Significant higher fluorescence intensity was measured in the ramp-side than flat-side, further confirming accumulation of MLC to the side of T cells contacting the ramp-like structures. A similar type of MLC-GFP accumulation on one side contacting the ramp-like structures was observed for CK636-treated T cells (11 out of 13 cells) as shown in Fig. 5C and Movie S3 in SI, but swift turns did not occur. MLC-GFP packets were not observed near the head-tail junction for the majority of ML7-treated T cells, and MLC-GFP fluorescence uniformly distributed within the cells (29 out of 40 cells) as shown in Fig. 5D and Movie S4 in SI, meaning that MLCK activity is essential for the accumulation of myosin II in head-tail junction regions of T cells. Taken together, contractile force generated near the head-tail junction regions of T cells via MLCK activity slowed down T cells and induced swift turns of T cells.

The formation of myosin II packets near head-tail junctions in crawling T cells has been previously observed^27^, but their roles in steering leading edge protrusion have not been clear. To further investigate the correlation between myosin II accumulation on one side of the head-tail junction and the turning behavior of T cells, MLC-GFP-transfected T cells were plated on flat substrates coated with ICAM-1 and time-lapse imaging was performed. A representative movie and time-lapse images are shown in Movie S5 in SI and Fig. 5E, respectively. To better visualize the correlation between the accumulation of MLC on one side of head-tail junctions and the turning direction, the outlines of T cells in the previous time-lapse images were overlaid. In most cases (30 out of 40), MLC clustering on one side coincided with turning to the same side within 25 sec, indicating that MLC accumulation on one side of the head-tail junctions regulates the migration direction. Frequently (28 out of 40), MLC clustering occurred in an alternating fashion (left → right → left, etc.), giving a zig-zag pattern of leading edge protrusion, resulting in directionally persistent migration.

### Lamellipodia formation and MLCK activity are critical for climbing sharp-edged structures

**Figure 6:**
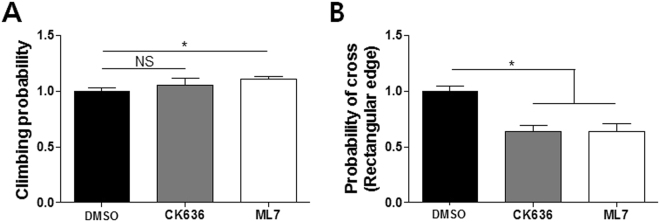
Effects of pharmacological inhibitors on relative climbing probability of T cells. (**A**) Relative climbing probability of DMSO-, CK636- and ML7-treated T cells climbing up the ramp-like structures. DMSO: *n* = 144; CK636: *n* = 113; ML7: *n* = 130. (**B**) Relative climbing probability of DMSO-, CK636- and ML7-treated T cells climbing up sharp-edged (rectangular edge) structures. DMSO: *n* = 159; CK636: *n* = 160; ML7: *n* = 159 (**B**). (Mann-Whitney U-test, two-tailed, NS: not significant, *p < 0.05).

Lastly, we asked whether the inhibition of lamellipodia formation and MLCK activity affected the overall climbing probability of T cells encountering the ramp-like structures. Time-lapse images of DMSO-, CK636-, and ML7-treated T cells encountering the ramp-like structures were analyzed to measure the probability of climbing. Relative climbing probability, the climbing probability of each treatment relative to DMSO-treatment, was calculated and plotted (Fig. 6A). While CK636-treatment exhibited minimal effects on T cells climbing the ramp-like structures, ML7-treatment slightly but significantly increased the climbing probability. In contrast to the ramp-like structure with smooth-slope changes used in the bulk of the experiments, when the sharp-edged structures fabricated by 0 min of baking shown in Fig. 1C were used for the identical experiments, CK636-, and ML7-treated T cells exhibited significantly reduced climbing probability compared to that for DMSO-treated T cells (Fig. 6B). These results suggest that the lamellipodia formation and MLCK-mediated contractility responsible for the intriguing turning behavior of T cells climbing the ramp-like structures have significant effects on the climbing efficiencies of T cells for sharp-edged structures with abrupt topography changes.

## Discussion

In this study, we investigated how T cells behave when they climb up the ramp-like structures with micron-scale topography. T cells climbing the ramp-like structures frequently turned to the perpendicular direction regardless of the incidence angle. Arp2/3-mediated lamellipodia formation and MLCK-mediated contractility were critical for the intriguing turning behaviors of T cells because T cells treated with pharmacological inhibitors for arp2/3 or MLCK no longer exhibited acute turning when they climbed the ramp-like structures. Dynamic imaging of MLC-GFP revealed that MLCK-mediated accumulation of MLC occurred at one side of head-tail junctions of T cells contacting the ramp-like structures prior to acute turning. Indeed, transient accumulation of MLC at one side of head-tail junction coincide with T cell turning to the side of MLC clustering for T cells on flat surfaces, indicating contractility near head-tail junctions in T cells somehow steer migration direction potentially by regulating local lamellipodia protrusion.

**Figure 7:**
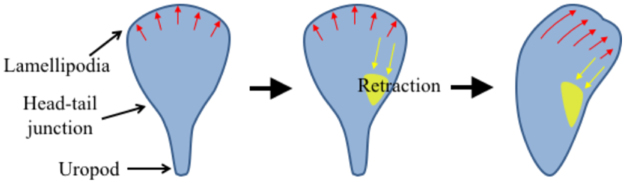
Model of T cell turning regulated by myosin light chain (MLC) accumulation near head-tail junction. Red arrows: leading edge protrusion, yellow patches: MLC, yellow arrows: contraction by MLC accumulation.

Detailed mechanisms of how myosin II-mediated contractility at one side of head-tail junction regulate local lamellipodia protrusion to induce turning need to be determined. Local activation of myosin II by MLCK can lead to local retraction of lamellipodia^30–32^ for strongly adhering cells such as endothelial cells and fibroblasts. Therefore, it is possible that MLC clustering at one side of T cells mediated by MLCK activity can cause retraction of lamellipodia at the same side, resulting in turning to the direction of MLC clustering as schematically shown in Fig. 7 ^33^. Indeed, pharmacological inhibitor study revealed that MLCK-mediated contractility slowed down migration speed of T cells encountering the ramp-like structures (Fig. 4B), and slightly reduced climbing probability (Fig. 6A), indicating local retraction of leading edge can be caused by MLCK. However, morphology and migration mode of T cells are completely different from those of fibroblasts and endothelial cells, thus roles of MLCK in regulating lamellipodia of T cells can also be different. Fibroblasts and endothelial cells strongly adhere on surfaces by forming focal adhesion complexes and generate lamellipodia with several micrometers long^30–32^, and MLCK-mediated contractility occurring ~5 μm behind the plasma membrane where lamellipodia and lamella interfaces retracts lamellipodia and enhances focal adhesion assembly^32^. In contrast to fibroblasts and endothelial cells, T cells weakly adhere on surfaces without forming focal adhesions, and generally generate relatively short lamellipodia with ~1 μm long.

Intriguing turning behavior of T cells climbing the ramp-like structures may directly contribute to search processes as turning angle regulation by class I myosin significantly enhance probability of finding rare antigen presenting cells in lymph nodes^34^. Alternatively, T cells made turns to efficiently climb up stiff topographical structures as shown in Fig. 6B, and turning angles themselves may not have important meanings for *in vivo* T cell trafficking. Abrupt topographical structures in local tissues may be formed during injury, edema by massive inflammation, and tissue remodeling during tumorigenesis. Considering that T cell receptor (TCR)-mediated direct cell-cell contacts are essential for T cell functions, climbing up stiff topographical structures within injured or inflamed tissues would be important for T cells to access local tissues to successfully eliminate pathogens and tumors.

## Materials and Methods

### Ramp-like structure fabrication

Periodic ramp-like structures were fabricated by the procedure schematically shown in Fig. 1. First, straight lines with rectangular cross-sections (width: 100 μm, height: 5 μm and interval between adjacent line patterns: 100 μm) were fabricated onto silicon wafers by standard photolithography processes based on SU-8 2 photoresist (Microchem) (Fig. 1A). Then, SU-8 2 photoresist patterns were baked at 150 °C for 1 min to induce reflow of patterned photoresist to form smooth ramp-like structures (Fig. 1B(i)). The ramp-like structures on silicon wafer were replicated twice using a UV-curable resin poly urethane acrylate (PUA) (Minuta Tech, Korea) by capillary force lithography (CFL)^26^ to generate identical structures of PUA on 18 mm-diameter of glass coverslips (Marienfeld) as previously described^23, 25^.

### Cell Culture

DO11.10 T cell receptor transgenic mice (Jackson Laboratories) bred in the POSTECH Biotech Center (PBC) were used to generate DO11.10 T cell blasts. All the T cell assay including mice treatments were performed in accordance with the guidelines approved by the Institutional Animal Care and Use Committee at PBC. DO11.10 blasts were obtained by stimulating cells in the spleen and lymph nodes of DO11.10 transgenic mice with 1 μg/ml of OVA323–339 peptide (ISQAVHAAHAEINEAGR, Peptron, Inc. Korea). Cells were cultured in RPMI 1640 (Invitrogen) containing 10% of FBS, 1% of penicillin-streptomycin and 0.1% of beta-mercaptoethanol (Sigma). 1–2 U/ml of IL-2 was added on the 2^nd^ day of stimulation and cells were used for experiments on the 5^th^ days. To inhibit arp2/3 complex, myosin light chain kinase (MLCK), ROCK and non-muscle myosin II, CK636 (100 μM, Sigma), ML7 (30 μM, Sigma), Y-27632 (50 μM, Sigma) and blebbistatin (50 μM, Sigma) were incubated with cells for 1 h, respectively. For arp2/3 complex inhibition, CK666, which is a more potent derivative of CK636^35^, can be used, instead.

### SEM sample preparation

DO11.10 T cells on substrates were fixed with 0.1 M cacodylate buffer (Sigma) containing 2.5% glutaraldehyde (Sigma) and 1% sucrose for 10 min at 4 °C. After rinsing samples with cacodylate buffer, they were post-fixed with 1% osmium tetroxide (Sigma) in cacodylate buffer for 20 min at room temperature, and extensively washed. Graded ethanol (30%, 50%, 70%, 80%, 90%, and twice in 99.5% ethanol for 5 min each) and isopentyl acetate (Sigma, at the rate of 1:3, 1:1, 3:1 with absolute ethanol and pure isopentyl acetate for 10 min each) were added onto samples. The samples in pure isopentyl acetate were completely dehydrated with a critical point dryer (Hitachi, hcp-2). Finally, the dried samples were coated with Pt by sputtering and SEM images were acquired.

### Live cell imaging

Ramp-like structures replicated with PUA on glass coverslips were treated with air plasma (200~500 w, Femto Science, Korea) and coated with ICAM-1 (10 μg/ml, PeproTech) for 1.5 h. After loading the ramp-like structures in Chamlide chambers (Live Cell Instrument, Korea) maintaining 37 °C and 5% of CO_2_, DO11.10 T cells in culture medium were added on the structures and time-lapse images of cells were acquired using an automatically-controlled microscope (Carl Zeiss Axio Observer.Z1) with 15-s interval for 20 min. The images were analyzed and processed with ImageJ (NIH).

### Visualization of myosin light chain (MLC)

To visualize MLC in T cells, MLC-GFP (gift of Michael F. Olson, University of Pennsylvania, US) plasmids were subcloned into a pMIG retroviral vector, and viruses were produced by transfection of phoenix cells using Lipofectamine 2000 (Life Technologies). For the infection of T cells with viruses, DO11.10 T cells harvested from the spleen and lymph node were mixed at the ratio of 1:2, added with 1 μg/ml of OVA323–339 peptide (ISQAVHAAHAEINEAGR, Peptron, Korea) and cultured in 24 well-plates at a concentration of 1 × 10^6^ cells/ml. On the 2^nd^ day of culture, each well was filled with viral supernatants, spun at 2000 RPM for 2 h and filled again with culture media containing 1–2 U/ml of IL-2. Then, the cells on 3^rd^ or 4^th^ day of culture were used.

### Statistical analysis

Statistical significance was tested using the Mann-Whitney U-test. For bar graphs, average values with standard error of mean (s.e.m.) are presented.

### Data availability

All data generated or analysed during this study are included in this published article (and its Supplementary Information files).

## Electronic supplementary material

Supplementary movie legends

Supporting Movie S1

Supporting Movie S2

Supporting Movie S3

Supporting Movie S4

Supporting Movie S5
