# Supplementary material for: Turning behaviors of T cells climbing up ramp-like structures are regulated by myosin light chain kinase activity and lamellipodia formation
Source: Sci Rep. 2017 Sep 14;7:11533. doi: 10.1038/s41598-017-11938-y (PMC5599526; doi:10.1038/s41598-017-11938-y)
Supplement: Supplementary file 1 — Supplementary movie legends [file 41598_2017_11938_MOESM1_ESM.pdf]

Turning behaviors of T cells climbing ramp-like structures are regulated by myosin light chain kinase activity and lamellipodia formation

Kwang Hoon Song,[1] *Jaehyun Lee,*[2] *Hong-Ryul Jung,*[2] *HyoungJun Park,*[1]

and Junsang Doh [1,2,*]

[1]Department of Mechanical Engineering, [2]School of Interdisciplinary Bioscience and Bioengineering (I-Bio), Pohang University of Science and Technology (POSTECH) San 31, Hyoja-dong, Nam-Gu, Pohang, Gyeongbuk, 790-784, Korea.

[*]Corresponding author:

Junsang Doh

Address: Pohang University of Science and Technology (POSTECH), San 31, Hyoja-dong Nam-Gu, Pohang, Gyeongbuk 790-784, Korea

Tel: +82-54-279-2189

Fax: +82-54-279-3199

E-mail: jsdoh@postech.ac.kr

Supplementary Information (SI)

Supplementary movie legends

Movie S1. A representative DIC movie of a T cell climbing ramp-like structures. Scale bar: 10 μm, Elapsed time: mm:ss.

Movie S2. A representative pseudo-color movie visualizing the relative intensities of MLC-GFP in a T cell climbing ramp-like structures. Scale bar: 10 μm, Elapsed time: mm:ss.

Movie S3. A representative pseudo-color movie visualizing the relative intensities of MLC-GFP in a CK636-treated T cell climbing ramp-like structures. Scale bar: 10 μm, Elapsed time: mm:ss.

Movie S4. A representative pseudo-color movie visualizing the relative intensities of MLC-GFP in a ML7-treated T cell climbing ramp-like structures. Scale bar: 10 μm, Elapsed time: mm:ss.

Movie S5. A representative pseudo-color movie visualizing the relative intensities of MLC-GFP in a T cell crawling on flat surfaces. Scale bar: 10 μm, Elapsed time: mm:ss.
